# Supplementary material for: How to measure work functions from aqueous solutions
Source: Chem Sci. 2023 Aug 29;14(35):9574–88. doi: 10.1039/d3sc01740k (PMC10498509; doi:10.1039/d3sc01740k)
Supplement: SC-014-D3SC01740K-s001 [file SC-014-D3SC01740K-s001.pdf]

Electronic Supplementary Material (ESI) for Chemical Science.
This journal is © The Royal Society of Chemistry 2023

Supporting Information

How to measure work functions from aqueous solutions

Michele Pugini[1], Bruno Credidio[1], Irina Walter[1], Sebastian Malerz[1], Florian Trinter[1,2], Dominik Stemer[1], Uwe Hergenhahn,[1] Gerard Meijer[1], Iain Wilkinson[3], Bernd Winter[1]*, and Stephan Thürmer[4]*

[1] Molecular Physics Department, Fritz-Haber-Institut der Max-Planck-Gesellschaft, Faradayweg 4-6, 14195 Berlin, Germany
[2] Institut für Kernphysik, Goethe-Universität, Max-von-Laue-Straße 1, 60438 Frankfurt am Main, Germany
[3] Institute for Electronic Structure Dynamics, Helmholtz-Zentrum Berlin für Materialien und Energie, Hahn-Meitner-Platz 1, 14109 Berlin, Germany
[4]Department of Chemistry, Graduate School of Science, Kyoto University, Kitashirakawa-Oiwakecho, Sakyo-Ku, 606-8502 Kyoto, Japan

Flow-Rate-Dependent Measurements of TBAI Aqueous Solutions

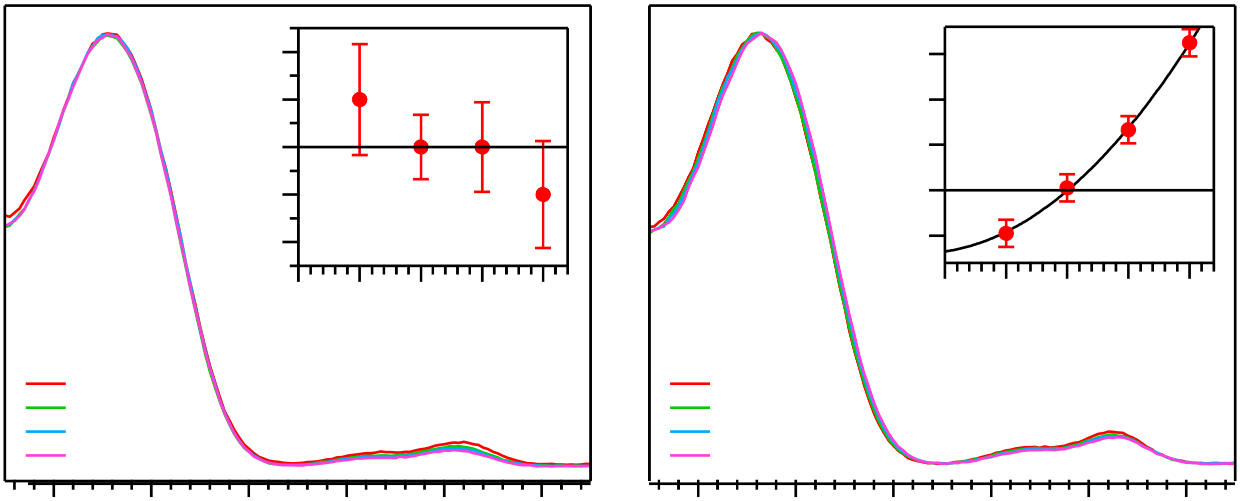

Figure S1: PE spectra of 12.5 mM **(A)** and 25 mM **(B)** $TBAI_{(aq)}$ showing the water $1b_1$ and I^- 5p PE features as a function of flow rate from 0.5-2.0 ml/min. Insets show the relative energy shift of the water $1b_1$ feature compared to 1 ml/min flow. The error bars are fit errors. The polynomial fit in the inset of B) is only a guide to the eye.

PE spectra were recorded from aqueous solutions injected into the spectrometer at various flow rates and with representative concentrations of 12.5 and 25 mM TBAI, to assess their residual streaming potentials. Any energy shift in the spectra as a function of liquid flow rate can be extrapolated to zero flow, at which the streaming potential must vanish. Alternatively, should there be no shift, the absence of a streaming potential can be confirmed for any flow rate. Figure S1 shows the PE spectra for 12.5 mM (A) and 25 mM (B) solutions for flow rates from 0.5-2.0 ml/min. All spectra were recorded at the P04 soft X-ray beamline[1] of the PETRA III synchrotron facility (Deutsches Elektronen-Synchrotron, DESY, Hamburg) at a photon energy of 401 eV and using the same spectrometer setup (*EASI*) and PtIr microplate assembly as in the laboratory experiments. The insets show the energy shift of the water $1b_1$ PE feature relative to the flow rate of 1 ml/min (dashed line), which

was utilized for all measurements presented in the main text. For 12.5 mM TBAI, no significant shift and thus no notable contribution of the streaming potential is observed. For 25 mM TBAI, while showing a clear trend towards higher energy shifts at higher flow rates, the difference between an (extrapolated) zero flow and 1 ml/min is below 15 meV. This gives us confidence that the contribution of the streaming potential is an insignificant fraction of the energy shifts discussed in the main text.

The Apparatus Fermi Level and Work Function

Measuring E_F from *metallic* systems is straightforward. Since electronic states are occupied in conductors up to E_F, the associated energy is revealed as a sharp, high-energy cutoff in PE spectra. In the absence of any disturbing potentials, the measured kinetic-energy value associated with E_F corresponds to $eKE_{EF} = h\nu - e\Phi_{det}$, with $e\Phi_{det}$ being the work function of the detection system. This somewhat counterintuitive result originates from the fact that photoelectrons experience the contact potential difference between the sample and apparatus, $\Delta e\Phi = e\Phi_{liq} - e\Phi_{det}$. The kinetic energy of the detected photoelectrons is thus modified by $\Delta e\Phi$, *i.e.*, $eKE_{EF} = h\nu - e\Phi_{liq} + \Delta e\Phi = h\nu - e\Phi_{det}$. For semiconductors and insulators – *i.e.*, condensed-phase systems exhibiting a band gap where the energy range around E_F is devoid of available states for electrons – E_F is not directly measurable. Similarly, liquid water does not exhibit electronic states in the vicinity of its Fermi level and can be modelled as a semiconductor with a direct band gap greater than 8 eV, where the exact value is still under debate.[2-4]

Note that the measured Fermi-level-feature kinetic energy, eKE_{meas}, is somewhat arbitrary, as it may be subject to experimental offsets associated with the detection system, such as a poorly calibrated energy scale. It is not an issue for Fermi referencing if the measured energy scale is linear, since all spectra are measured with the same device and are thus subject to the same systematic measurement offsets, *i.e.*, only relative kinetic energies are involved. This is the case for our hemispherical electron analyzer; care should be taken with non-linear systems such as time-of-flight analyzers, though. Previously, we corrected eKE_{meas} with the offset factor for our system, determined as 0.224 ± 0.008 eV, which yielded $e\Phi_{det} = 4.293 \pm 0.009$ eV.[5] In the present experiments, we measured the 3p peak of Argon gas after the LJ experiments and compared it with the reference value of 15.759735 ± 0.000001 eV[6] to confirm the energy-scale correction. This yielded a consistent $e\Phi_{det} = 4.30 \pm 0.04$ eV; the somewhat larger error originates from observed fluctuations in the Ar 3p peak positions over multiple days.

Pitfalls and Considerations for Accurate Fermi Referencing

While Fermi-referenced measurements seem straightforward, if streaming potentials and any other extrinsic potentials have been considered or eliminated, grounded measurements are prone to interferences from sub-optimal experimental conditions. Reliable measurements correspondingly necessitate the utmost care. We encountered several systematic experimental errors during our studies, which can negatively affect the measurement. Here, we give a brief overview of the associated potential pitfalls.

1) In the main text, it was noted that a consistent value was measured for the Fermi edge of our reference metal samples ($eKE_{EF} = 36.30$ eV), and that this value is stable, as it is an intrinsic property of our apparatus. We observed, however, that this value can be temporarily altered slightly – on the order of 50 meV or more – when measuring certain samples, such as solutions containing organic compounds. This may stem from persistent surface-adsorption layers on the inner walls of the apparatus, which desorb over the course of days

or even weeks. We correspondingly recommend the constant monitoring of the Fermi-edge position when attempting to measure Fermi-referenced spectra.

2) Another important aspect in LJ-PES is the continuous evaporation from the liquid surface, which forms some rather ill-defined adsorbate layer on all inner walls of the apparatus. This alters the surface potentials of the interaction chamber, which causes spectral shifts over time and with varying chamber pressure; for example, a shift of several hundred meV has been observed over a 2–4-hours period after starting experiments.[5]

3) In the case of the glass-nozzle assembly, grounding was achieved via metallic inset tubes placed in between the HPLC pump and PEEK liquid delivery line prior to injection into the vacuum chamber. As flow characteristics of the solution in this metallic inset are altered, crystallization of solutes may occur, which deteriorates the grounding. We observed shifts on the order of ~100 meV of all PE spectra in such a case. Cleaning and reassembling the liquid delivery system recovered the original energetic positions of PE spectra.

4) Figure S3 compares PE spectra from the PtIr microplate, a new, passivated glass nozzle, and a glass nozzle after running solutions with a particularly high (or low) pH value. Large shifts (up to ~300 meV) of all PE spectra were observed in the latter case. The unshifted PE spectra could not be recovered, even after several days. It is very likely that the surface properties of the inner glass-nozzle walls were chemically altered, which could lead to a significant streaming potential, or alternative additional unwanted potentials. Furthermore, the metallic inset tube used to ground the solutions may have been corroded. Thus, we found that the glass-nozzle assembly is more prone to detrimental effects, and measurements from a PtIr microplate (or other nozzles which provide both proper grounding and chemical resistance) are preferred.

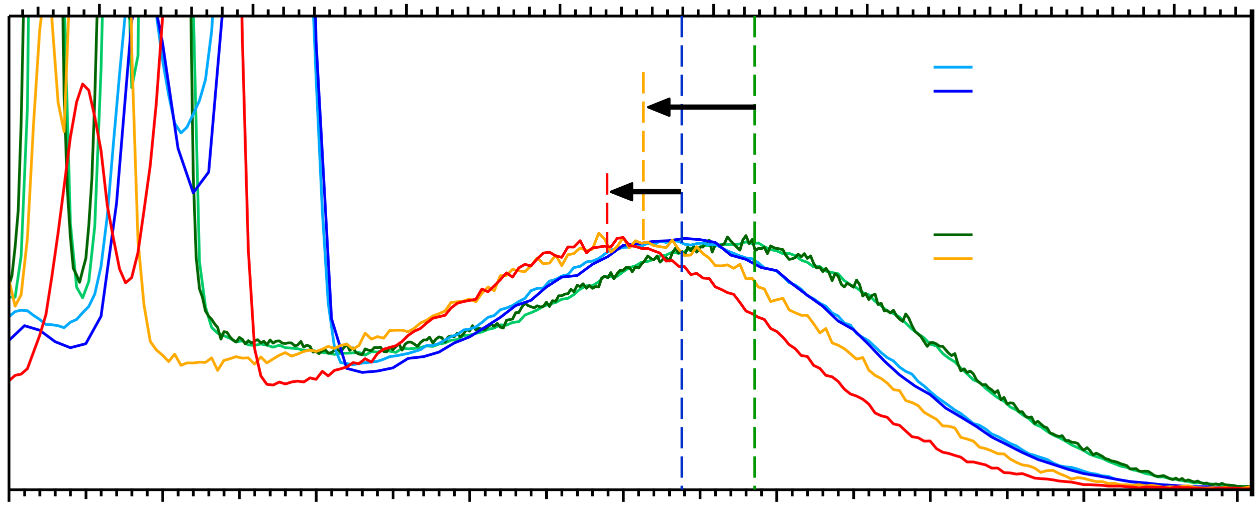

Figure S2: Exemplary valence PE spectra of reference water (with 50 mM NaCl added) and 25 mM $TBAI_{(aq)}$, measured from a grounded liquid jet with hν = 40.814 eV. If properly prepared, PE spectra measured from a PtIr microplate are equivalent to the ones from a glass capillary for both reference water (light and dark blue curves) and $TBAI_{(aq)}$ (light and dark green curves). However, the PE spectra shifted considerably after running solutions with a pH value well removed from 7; both a pH close to 0 and 14 was reached before the spectra shown here were measured. Specifically, shifts of ~250 meV and 310 meV were observed for reference water (red curve) and 25 mM $TBAI_{(aq)}$ (yellow curve), respectively. We noticed such shifts whenever solutions with particularly

high or low pH values were measured prior to Fermi-referenced measurements with glass-capillary nozzles, suggesting a pH-induced deterioration of the glass and an associated change in liquid streaming potential.

Additional Figures

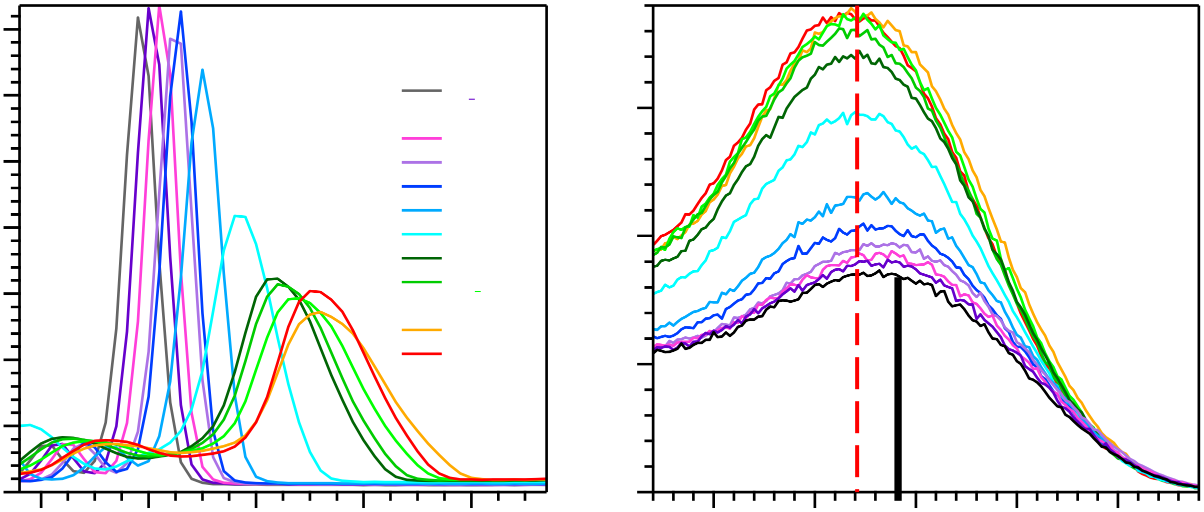

Figure S3: Close-up on the **A)** gas and **B)** liquid $1b_1$: The data from Fig. 4 in the main text are shown without intensity normalization and vertical offsets, *i.e.,* here, the PE intensities are shown as-measured.

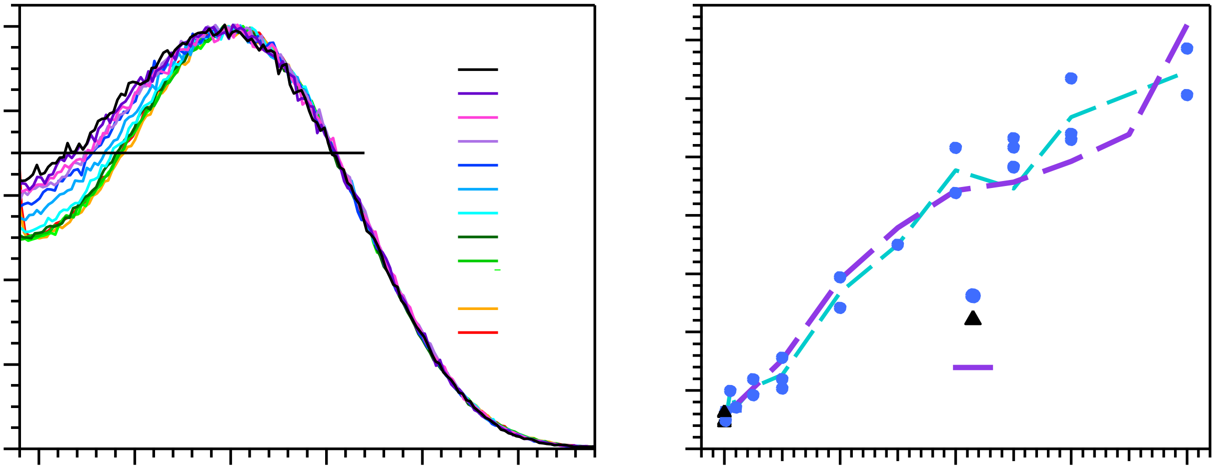

Figure S4: **A)** Liquid $1b_1$ band shape as a function of TBAI concentration; the spectra are the same as in Figs. 4 and S3. Spectra have been normalized to unit height at the $1b_1$ band and shifted to maximum overlap at the high-energy shoulder to emphasize the change in shape. **B)** Values of the peak width as extracted from A) as a function of TBAI concentration. The widths were taken from the $1b_1$ band at 70% signal height (dashed line in panel A) and do not originate from the fits discussed in the main text. Fit results (FWHM values) from our previous study[7]

have been added for comparison; this data has been arbitrarily scaled by a factor of 3 and aligned to the reference water value.

In the main text, it was explained that the liquid water $1b_1$ band was fitted by a single peak over a limited data range because of overlapping features from the gas phase, which made a full fit of the spectrum difficult. Thus, it was not possible to extract a peak width from the fit, due to the constrained nature of the procedure. Instead, in Fig. S4, we plot the total observed width of the PE band in the data directly at 70% band height, a value arbitrarily chosen to avoid interference from the signals of the nearby orbitals and the gas phase. The result in Fig. S4B serves as a confirmation of the overall trend of a broadening liquid-water $1b_1$ band, as observed in our previous study,[7] which is also replicated as a purple dashed line. This confirms that the shape of the liquid bands is the same, both in the grounded and biased measurements. Any presence of V_{tot} does not lead to additional broadening, since, similarly to the biased case, this potential leads to a rigid shift of all liquid features. This is furthermore consistent with the observed excellent match of biased and unbiased PE spectra; see, for example, Fig. 7A in the main text.

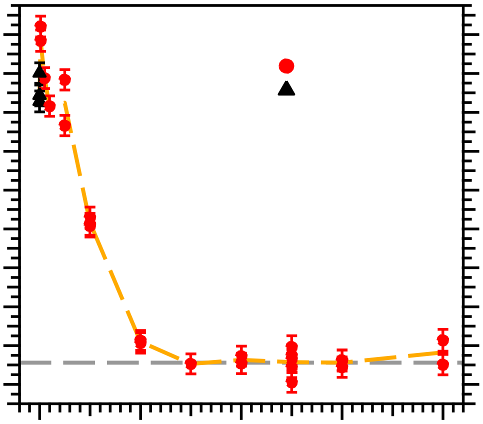

Figure S5: Peak width (FWHM) of the gas-phase $1b_1$ peaks as a function of TBAI concentration from peak fits to the PE spectra. Error bars represent the quadratic addition of fit errors (one sigma) and a general uncertainty in the measurement of 5 meV. Red dots indicate results from $TBAI_{(aq)}$ (5 mM NaCl have been added for concentrations at and below 1 mM TBAI to assure sufficient conductivity) and black triangles indicate reference water results (no TBAI but 50 mM NaCl). The minimal FWHM (indicating $|V_{tot}| \approx 0$ V) is reached from about ~10 mM up to the highest concentration. The constant value of ~70 meV indicates that any changes are below our ability to measure peak widths in our experiment.

References

1. J. Viefhaus, F. Scholz, S. Deinert, L. Glaser, M. Ilchen, J. Seltmann, P. Walter and F. Siewert, *Nucl. Instrum. Methods Phys. Res., Sect. A*, 2013, **710**, 151-154.
2. F. Williams, S. P. Varna and S. Hillenius, *J. Phys. Chem.*, 1976, **64**, 1549.
3. A. Bernas, C. Ferradini and J.-P. Jay-Gerin, *Chem. Phys.*, 1997, **222**, 151-160.
4. C. G. Elles, A. E. Jailaubekov, R. A. Crowell and S. E. Bradforth, *J. Chem. Phys.*, 2006, **125**, 044515.
5. S. Thürmer, S. Malerz, F. Trinter, U. Hergenhahn, C. Lee, D. M. Neumark, G. Meijer, B. Winter and I. Wilkinson, *Chem. Sci.*, 2021, **12**, 10558-10582.
6. I. Velchev, W. Hogervorst and W. Ubachs, *J. Phys. B: At., Mol. Opt. Phys.*, 1999, **32**, L511-L516.
7. B. Credidio, M. Pugini, S. Malerz, F. Trinter, U. Hergenhahn, I. Wilkinson, S. Thürmer and B. Winter, *Phys. Chem. Chem. Phys.*, 2022, **24**, 1310-1325.
